# Reviewed article: Cruz GL, Andrade GC, Rauber F, Levy RB, Louzada MLC. Application of the Nova food classification in the 2017-2018 Family Budget Survey: a strategy for monitoring adherence to the recommendations of the Food Guide for the Brazilian Population. Epidemiol Serv Saude. 2025;34:e20240369

**DOI:** 10.1590/S2237-96222025v34e20240369.b

**Published:** 2025-05-12

**Authors:** Raquel de Deus Mendonça

**Affiliations:** Universidade Federal de Ouro Preto

**Table:** 

Rating	Excellent	Good	Below Average	Poor
Interest				✓
Quality			✓	
Originality				✓
Overall			✓	

**Table:** 

Questionnaire	Yes	No	Not applicable
Does the manuscript contain new and significant information to justify publication?		✓	
Does the Abstract (Summary) clearly and accurately describe the content of the article?	✓		
Is the problem significant and concisely stated?		✓	
Are the methods described comprehensively?	✓		
Are the interpretations and conclusions justified by the results?	✓		
Is adequate reference made to other work in the field?	✓		

## Manuscript Structure:

Length of article is: Too short

Number of tables is: Adequate

Number of figures is: Adequate

Please state any conflict(s) of interest that you have in relation to the review of this paper (state “none” if this is not applicable): None

## Comments

Trata-se de um relato de aplicação da classificação NOVA dos alimentos com dados de consumo alimentar da Pesquisa de Orçamentos Familiares 2017-2018. O manuscrito relatada detalhadamente as etapas da aplicação da NOVA nos dados dos itens alimentares derivados do Recordatório 24 horas. Por fim, apresenta a possibilidade de usar a classificação NOVA com pequenas diferenças entre avaliados com pequenas incertezas.

Apresento para este relato as seguintes preocupações:

- Qual a hipótese do estudo?

- Apesar da descrição das etapas, observo a impossibilidade de replicação do método, devido há não clareza dos seguintes aspectos:

a) Quais as fontes das consultas externas (page 5, linha 4-5)? Quais bases de dados externas (page 5, linha 6-7)? Quais sites? Como esses sites foram selecionados?

b) Como foi definida “maior ocorrência” (page 5, linha 9)

c) Quais os critérios para seleção dos alimentos do quais realizou-se o levantamento das listas de ingredientes (page 6, linha 25-30)

d) Em relação a frase a seguir “Características da cultura e hábitos alimentares brasileiros também foram consideradas na tomada de decisão, optando pela alternativa mais habitual para a classificação.”. Como definidos as características da cultura e hábitos alimentares? O que seria alternativa mais habitual?

- Para as análises sugiro aplicação de testes de concordância.

- Há diferença entre os termos industrializados, ultraprocessados, produtos prontos industrialmente fabricados? Se sim, sugiro inserir na Introdução.

- Padronizar o uso de “extensão e o propósito do processamento” em substituição ao grau de processamento.

- Há frases e parágrafos sem citação das referências, exemplo: (page 3, linha 3 a 14); (page 3, linha 44 a57);

- Rever a redação da frase a seguir para aperfeiçoar a clareza: “Verificou-se os grupos da Nova aos quais pertenceriam os ingredientes de um item alimentar elegível para desagregação caso fosse desagregado, dispensando a necessidade de desagregação caso todos pertencessem ao mesmo grupo.”

Perante essas fragilidades metodológicos recomendo a rejeição do manuscrito.

